# Synaptic depression and short-term habituation are located in the sensory part of the mammalian startle pathway

**DOI:** 10.1186/1471-2202-7-38

**Published:** 2006-05-09

**Authors:** Nadine S Simons-Weidenmaier, Maruschka Weber, Claudia F Plappert, Peter KD Pilz, Susanne Schmid

**Affiliations:** 1 Tierphysiologie, Zoologisches Institut, Fakultät für Biologie, Universität Tübingen, Auf der Morgenstelle 28, 72076 Tübingen, Germany; 2 Physiologisches Institut, Universität Würzburg, Röntgenring 9, 97070 Würzburg, Germany

## Abstract

**Background:**

Short-term habituation of the startle response represents an elementary form of learning in mammals. The underlying mechanism is located within the primary startle pathway, presumably at sensory synapses on giant neurons in the caudal pontine reticular nucleus (PnC). Short trains of action potentials in sensory afferent fibers induce depression of synaptic responses in PnC giant neurons, a phenomenon that has been proposed to be the cellular correlate for short-term habituation. We address here the question whether both this synaptic depression and the short-term habituation of the startle response are localized at the presynaptic terminals of sensory afferents. If this is confirmed, it would imply that these processes take place prior to multimodal signal integration, rather than occurring at postsynaptic sites on PnC giant neurons that directly drive motor neurons.

**Results:**

Patch-clamp recordings *in vitro *were combined with behavioral experiments; synaptic depression was specific for the input pathway stimulated and did not affect signals elicited by other sensory afferents. Concordant with this, short-term habituation of the acoustic startle response in behavioral experiments did not influence tactile startle response amplitudes and vice versa. Further electrophysiological analysis showed that the passive properties of the postsynaptic neuron were unchanged but revealed some alterations in short-term plasticity during depression. Moreover, depression was induced only by trains of presynaptic action potentials and not by single pulses. There was no evidence for transmitter receptor desensitization. In summary, the data indicates that the synaptic depression mechanism is located presynaptically.

**Conclusion:**

Our electrophysiological and behavioral data strongly indicate that synaptic depression in the PnC as well as short-term habituation are located in the sensory part of the startle pathway, namely at the axon terminals of sensory afferents in the PnC. Our results further corroborate the link between synaptic depression and short-term habituation of the startle response.

## Background

The mammalian startle response is a protective response that results in the contraction of skeletal and facial muscles in response to a sudden acoustic, tactile or vestibular stimulus. This response is modulated by elementary forms of learning such as sensitization [1,2] and habituation [3,4]. Short-term habituation is an attenuation of the startle response upon repeated presentation of startle stimuli within one session that is reversible within several minutes [5,6]. The degree of attenuation varies between different animal species and different mouse strains [7,8].

**Figure 1:**
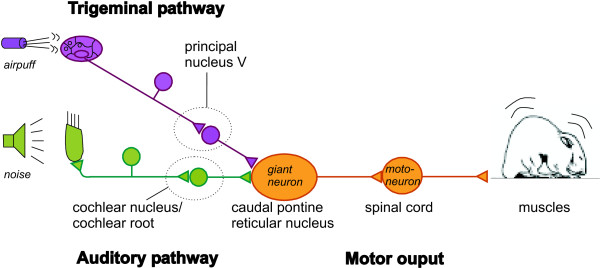
**Scheme of the primary startle pathway in rats and mice**. Acoustic or tactile information is conveyed by only two serial types of sensory neurons in each pathway to the caudal pontine reticular nucleus (PnC). Here, synapses of secondary sensory neurons converge onto giant neurons that form the sensorimotor interface of the startle response. Axons of the PnC giant neurons directly project onto motorneurons in the facial nucleus or spinal cord. In the electrophysiological experiments of the present study, axons of the secondary sensory neurons were stimulated by extracellular electrodes, while patch clamp recordings were performed from PnC giant neurons in rat and mouse brain slices.

The startle response is mediated by a short, well-described primary pathway [for review see [9]], which is depicted in figure 1. A relatively small population of giant neurons in the PnC represents the sensorimotor interface of the startle pathway [10-20]. These neurons receive multiple sensory inputs converging from auditory, trigeminal and vestibular afferent pathways [[21,22] see [9,23,24] for reviews] and their axons project directly onto motoneurons that innervate the facial and skeletal muscles [18]. Previous behavioral and pharmacological studies have indicated that the mechanism responsible for short-term habituation of the startle response must be located at the synapses within the PnC [5,6,18,25-27].

We have previously shown that repeated short trains of action potentials (closely mirroring the activity of sensory afferent fibers during the presentation of a startle stimulus *in vivo*) induce an exponential decay of the synaptic response amplitudes in PnC giant neurons in rat brain slices. This form of synaptic depression was proposed to be the neural correlate for short-term habituation of the startle response [22,28]. In the present study, we address the question whether the underlying mechanism for synaptic depression is localized at presynaptic sites belonging to the sensory branch of the startle pathway or rather at postsynaptic sites on the PnC giant neurons that directly activate motoneurons. This distinction is critical, since if synaptic depression occurs presynaptically, it would take place before multimodal integration of startle stimuli, whereas postsynaptic synaptic depression should be general for all sensory input. We performed patch-clamp recordings of PnC giant neurons in rat and mouse brain slices during stimulation of auditory and trigeminal afferent pathways. The depression induced by individual stimulation of these pathways in brain slices from rats and mice was examined for any possible interference arising from stimulation of the other pathway and the ensuing depression there. In parallel, behavioral experiments were performed with mice of the same strain used *in vitro *to investigate whether there is interference between short-term habituation to acoustic and tactile startle stimuli. Further electrophysiological studies included analysis of the passive membrane properties of PnC neurons and of short-term plasticity before and during synaptic depression. Additionally, the dynamic properties of the stimulated synapses were examined to provide more insight about the location of the synaptic depression mechanism.

## Results

**Figure 2:**
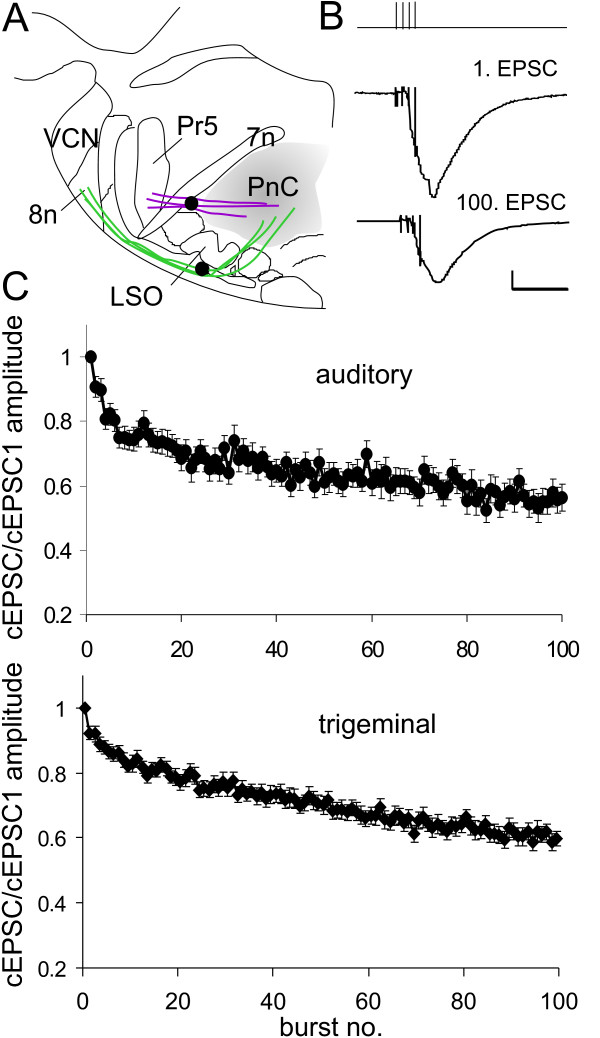
**Synaptic depression in the PnC**. A: Scheme of a rat brainstem slice at bregma -9.8 and interaural -0.8. Fibers of the secondary auditory neurons (emerging e.g. from the root of the 8^th ^nerve and/or the VCN) and from trigeminal neurons (emerging from the most ventral part of the Pr5) are plotted. Filled circles indicate the positions of the concentric bipolar extracellular stimulation electrodes. VCN: ventral cochlear nucleus, LSO: lateral superior olive, 8n: root of the auditory nerve, 7n: 7th nerve, Pr5: principal nucleus of the 5th nerve, PnC: caudal pontine reticular nucleus. B: Stimulation of auditory afferents with a short train of four pulses (within 12 ms) evoked a compound EPSC (cEPSC) in PnC giant neurons of rats, since single responses strongly summated. The maximum amplitude of the cEPSC decreased upon repeated activation. Original traces of the first and the 100^th ^cEPSC of one sequence are shown; the horizontal bar indicates 25 ms, the vertical bar 50 pA. C: cEPSC amplitudes in PnC giant neurons evoked by 100 bursts applied to auditory fibers (*top*) and trigeminal fibers (*bottom*) in rat slices exponentially decreased to 56 % and 60 % of the respective initial value (n = 29 for auditory and n = 44 for trigeminal stimulation). cEPSCs for each cell were normalized to the first evoked cEPSC of the respective cell.

Auditory and trigeminal inputs have been shown to converge on PnC giant neurons (see Fig. 1) and both undergo homosynaptic depression when repeatedly stimulated by presynaptic bursts [22,28]. To induce synaptic depression, auditory and trigeminal afferent fibers were stimulated with 150 μs pulses applied by concentric extracellular electrodes placed either in the auditory stria ventral to the lateral superior olive (LSO, Fig. 2A) or medial to the ventral portion of the principal nucleus of the 5^th ^nerve (Pr5, Fig. 2A) in rat and mouse brain slices. Four stimulus pulses delivered within 12 ms made up each burst and 100 bursts were applied at frequencies of either 1 or 0.1 Hz. The burst stimulation elicited short trains of action potentials in the afferent sensory fibers that should resemble the normal neuronal activity (high frequency firing) in these fibers during the presentation of an acoustic or tactile startle stimulus. The acoustic stimuli used in behavioral studies are typically sudden, loud noise pulses (>80 dB SPL) of about 10 to 30 ms duration and presented at frequencies between 0.1 and 0.03 Hz.

The postsynaptic currents evoked by the single pulses within one burst summated strongly due to the large membrane time constant of giant neurons. Thus, burst stimulation of sensory afferents evoked compound synaptic responses in PnC neurons (see Fig. 2B) that have been shown to be mediated by ionotropic glutamate receptors [28]. Peak amplitudes of the compound postsynaptic excitatory currents (cEPSCs) were measured with whole-cell patch clamp recordings at -70 mV. During a sequence of 100 bursts applied to sensory afferent fibers at a frequency of 1 Hz, the cEPSC peak amplitudes decreased to 56 ± 3 % of the initial response evoked when auditory fibers were stimulated (means of cEPSC amplitudes 91–100, n = 29) and dropped to 60 ± 2 % of the initial amplitude when trigeminal fibers were stimulated (n = 44, see Figs. 2B and 2C and). The depression of the cEPSCs lasted for about 10 minutes before amplitudes recovered to the initial value (data not shown, see [22,28]). Resting membrane potentials and input resistances of the postsynaptic neurons were analyzed during the recordings. The resting potential was -54.7 ± 1.53 mV before and -53 ± 1.42 mV immediately after depression of burst responses was induced by 100 auditory burst stimuli at 1 Hz (n = 17). The input resistance of the recorded cells was 100 ± 17 MΩ before and 114 ± 19 MΩ during depression (n = 22), as assessed by the I/O function of current responses to three small hyperpolarizing voltage pulses. This shows that the postsynaptic cell parameters were quite stable during recordings and that there were no alterations in postsynaptic membrane permeability at or near rest during depression.

### Interaction of auditory and trigeminal input in the PnC

**Figure 3:**
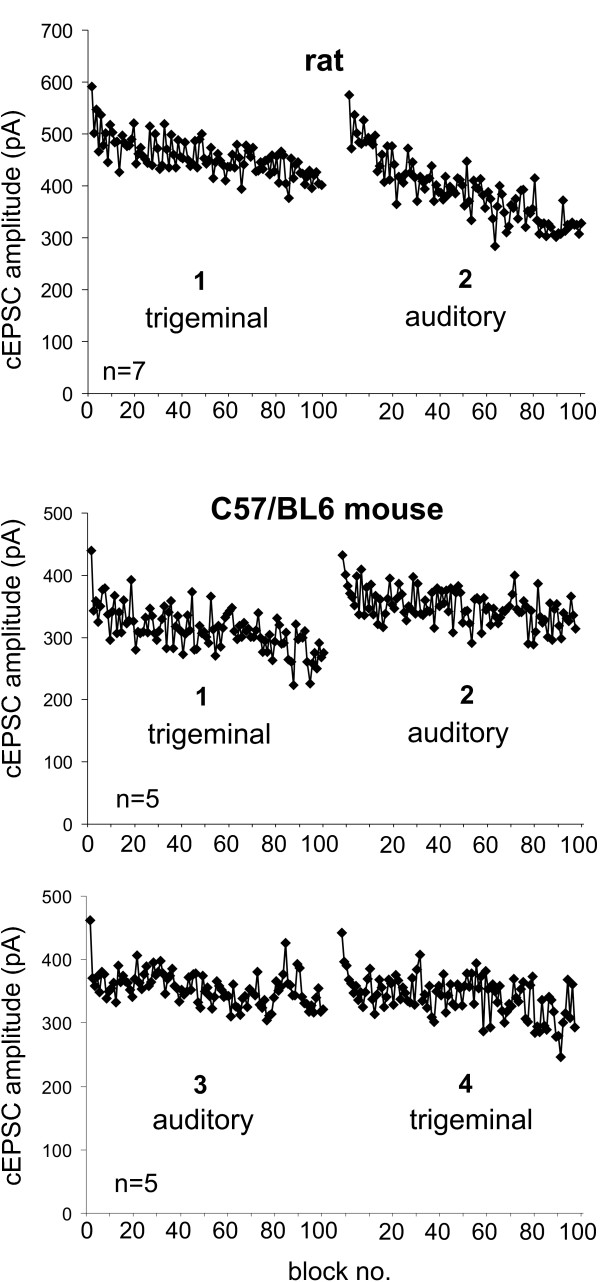
**Synaptic depression of auditory fibers does not interfere with synaptic depression of trigeminal afferents**. A: 100 bursts were applied at 1 Hz first to the trigeminal and immediately afterwards to the auditory fibers in rats (n = 7). Both burst sequences induced considerable synaptic depression in the respective pathway (general linear model: p < 0.0001), showing that the induction of synaptic depression in one pathway did not interfere with subsequent synaptic depression in the other pathway. B: This protocol was repeated in C57/BL6 mouse slices (n = 5). Again, both sequences induced synaptic depression in the respective pathway. C: After a recovery period, both sequences of 100 bursts were again applied, but first to the auditory and then to the trigeminal pathway. The first trigeminal cEPSCs of sequences 1 and 3 as well as the first auditory cEPSCs from sequences 2 and 4 were not statistically different.

If synaptic depression is due to a presynaptic mechanism located at the sensory afferent neuron terminals, synaptic depression in one sensory pathway should not affect synaptic transmission in the other pathway. We tested this hypothesis in rats and C57BL/6 mice. Presynaptic stimulation intensity was adjusted until similar cEPSC amplitudes were elicited by stimulation in either pathway. 100 bursts were first applied to the trigeminal pathway and then immediately thereafter to the auditory afferent fibers. Figure 3 (*top *and *middle*) shows that repeated trigeminal stimulation in rats and mice induced an exponential decay of cEPSC amplitudes (sequence 1). The auditory stimulation following again led to an exponential decay (sequence 2). In mice, we additionally reversed the order of the pathway stimulation after a 15 min recovery period (*bottom*, sequences 3 and 4). In summary, each 100 burst sequence induced a significant cEPSC decay (general linear model: F(1,490–881) ≥ 21.49, p < 0.0001). Moreover, there was no significant difference between the first trigeminal cEPSCs of sequences 1 and 3 or between the first auditory cEPSCs from sequences 2 and 4 (t(4) ≤ 0.035, p ≥ 0.48), indicating that synaptic depression in one pathway inhibited neither synaptic transmission in the other pathway nor subsequent synaptic depression.

### Interaction of acoustic and tactile startle habituation

**Figure 4:**
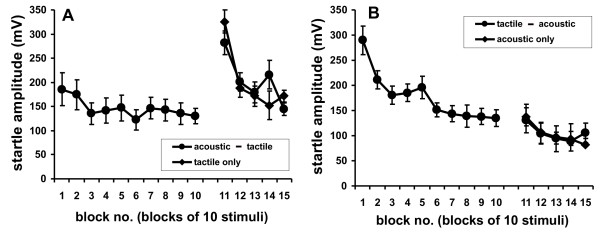
**Habituation of the startle responses to tactile and acoustic stimuli**. A: Tactile startle response of C57BL/6 mice (blocks 11–15: each block is the mean response to 10 stimuli) following either habituation to 100 acoustic startle stimuli ("acoustic→tactile" *filled circles*, displayed as 10 blocks of mean responses to 10 stimuli) or presentation of 50 dB SPL background noise only ("tactile only" controls, *diamonds*). B: Acoustic startle response (blocks 11–15) following either 100 tactile stimuli ("tactile→acoustic", *filled circles*) or presentation of 95 dB SPL background noise only ("acoustic only" controls,*diamond*s; n = 12).

Given that synaptic depression is the mechanism underlying behavioral short-term habituation, habituation should also be specific to the modality used to induce it. In other words, habituation to acoustic stimuli should not affect the startle response to tactile stimuli, and vice versa. We tested this hypothesis in C57/BL6 mice. In C57/BL6 mice, both the acoustic and tactile startle responses decreased significantly (Fig. 4, blocks 1–10; general linear model: F(1,107) ≥ 8.92, p ≤ 0.0035). However, habituation to tactile startle stimuli was much stronger than habituation to acoustic stimuli in this mouse strain. In both cases, habituation had no influence on the startle response to subsequent stimuli of a different modality. The tactile startle response was the same in the group with preceding acoustic stimulation (group "acoustic→tactile") and the control group with no previous stimulation (group "tactile only"; block 11 difference: t(22) = 0.31, p = 0.76; difference of means of blocks 11–15: t(22) = 1.84, p = 0.080; Fig. 4). The course of the acoustic startle response was also the same whether or not tactile stimuli were presented before the acoustic stimuli (block 11 difference: t(22) = 0.22, p = 0.83; difference of means of blocks 11–15: t(22) = 0.07, p = 0.94).

In summary, the data shows that both synaptic depression and short-term habituation are pathway/modality specific. This also corroborates the hypothesis that short-term habituation of the startle response is associated with synaptic depression in the PnC.

### Depression of burst responses is accompanied by altered short-term plasticity

**Figure 5:**
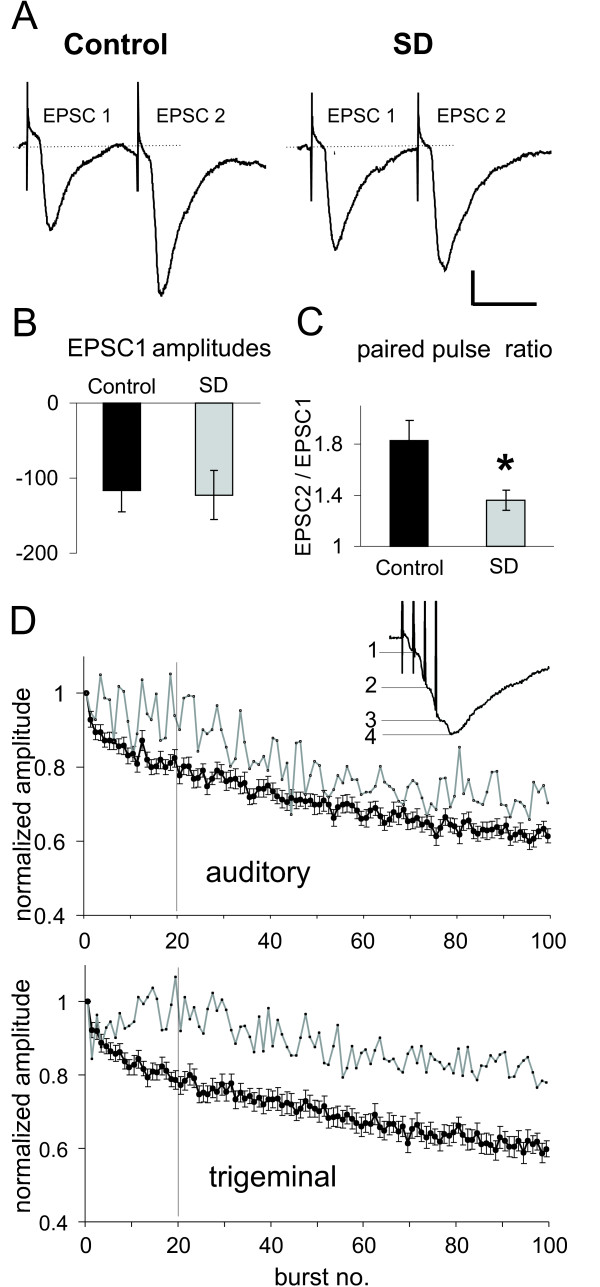
**Short-term plasticity is altered during synaptic depression**. A:Paired pulses applied with 50 ms interstimulus intervals (ISI) to the auditory pathway in rat brain slices revealed paired pulse facilitation (ppf). Typical EPSC traces evoked by paired pulses (with EPSC1 and EPSC2) in a PnC giant neuron under control conditions (control) and during synaptic depression (SD), immediately after application of a 100 burst sequence. Scale bars: vertical 30 pA, horizontal 30 ms. B: Mean EPSC1 amplitudes before (control) and during synaptic depression (SD). Measurements were repeated 10 times at 1 Hz and traces were subsequently averaged for each cell. The absolute EPSC1 amplitude was not changed by synaptic depression (n = 17). C: Mean paired pulse ratio (EPSC2/EPSC1) of paired pulses before (control) and during synaptic depression (HSD). The paired pulse ratio was significantly reduced during synaptic depression (n = 17). Error bars indicate S.E.M. D: Analysis of the amplitudes of the first response (gray line) within the cEPSCs elicited by 100 bursts revealed that these did not decay as much as the overall cEPSC amplitudes (filled circles) in both auditory (*top*) and trigeminal (*bottom*) synapses. During the first 20 bursts, there was no decay of the first response at all, whereas cEPSC amplitudes exponentially declined. This implicates that predominately late responses within the cEPSC are reduced, and that this reduction accounted for most of the decline in overall cEPSC amplitudes. The inset shows a typical cEPSC trace, where the single responses could be distinguished.

The pathway specificity of synaptic depression may indicate that a presynaptic mechanism is the underlying factor. However, a local postsynaptic mechanism restricted to the activated postsynaptic sites (e.g. glutamate receptor desensitization) could also account for pathway specificity. A feature that is commonly associated with presynaptic modulation of synaptic efficacy is an alteration of short-term plasticity. In the following experiment, we used a common paired-pulse paradigm to test whether short-term plasticity changed during synaptic depression. Pairs of pulses with an interstimulus interval (ISI) of 50 ms were applied before (control) and immediately after a sequence of 100 bursts (depressed, HSD). The single EPSCs evoked by each of the pulses were clearly separated from one another (Fig. 5A). Only ten traces were averaged for each condition to ensure that synapses in the "depressed" state could not recover during paired pulse measurements. There was little variability in the EPSC amplitudes within one condition and the amplitude of EPSC1 did not change during 10 repetitions of the paired pulse protocol (1^st ^versus 10^th ^EPSC1: t(15) = 0.49, p = 0.315), showing that paired pulses themselves did not induce synaptic depression. The paired pulse ratio was determined for each cell from the averaged trace. Under control conditions, EPSC2 amplitudes were always substantially larger than EPSC2 amplitudes by a mean factor of EPSC2/EPSC1 = 1.83 ± 0.16 (n = 17 cells, Fig. 5A). Surprisingly, the mean amplitude of EPSCs1 in the depressed state directly after the application of 100 burst stimuli did not decrease (-116.6 ± 28.3 pA before and -122.7 ± 33.1 pA during depression, n = 17, Fig. 5B), whereas the mean amplitude of the second EPSCs decreased from -184.9 ± 42.7 pA to -160.8 ± 43.4 pA, revealing a significantly reduced mean paired pulse ratio of EPSC2/EPSC1 = 1.36 ± 0.08 during depression (t(16) = 3.36, p = 0.002, Fig. 5c). In summary, this shows that paired pulses themselves did not induce synaptic depression and that the depression induced by 100 bursts was accompanied by alterations in the paired pulse ratio. Moreover, responses to single pulses did not seem to be depressed after 100 bursts, instead the alteration in short-term plasticity as assessed by the reduced paired pulse facilitation may account for much of the attenuation of the compound EPSC amplitude resulting from a burst stimulus. In other words, the above experiments indicate that the depression of cEPSC amplitudes is not due to general depression of synaptic transmission, but is instead the result of alterations in short-term plasticity that specifically lead to an attenuation of responses to stimuli late within a burst. We verified this by analyzing the burst responses evoked by the 100 bursts in more detail. The amplitude of the first response within one burst was measured in all cells with clearly distinguishable single responses within one burst (see inset, Fig. 5D). It was found that the first responses were not as strongly depressed as the overall cEPSC amplitudes. In figure 5D, the cEPSC amplitudes and the amplitudes of the initial response within each burst are plotted for each pathway. In 30 cells the first response declined to 71% of the starting amplitude at auditory synapses but to only 81% at trigeminal synapses, whereas the overall depression of cEPSCs in the same cells was 61% in both pathways. Moreover, depression of the first response started only after about 20 bursts, indicating that short-term depression within one burst increases with repeated burst stimulation and that this accounts for the majority of the attenuation of cEPSC amplitudes.

### Multiple stimuli at high frequency are necessary to induce synaptic depression

**Figure 6:**
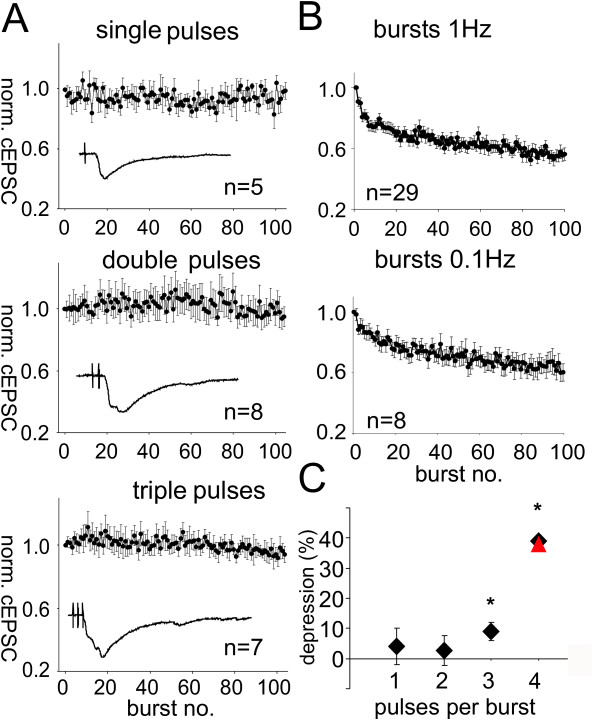
**Repeated high frequency stimulation is required for synaptic depression in the PnC**. A: Stimulation of PnC giant neurons with 100 single, double, or triple pulses with 4 ms ISI applied at 1 Hz to auditory fibers in rat brain slices. All cEPSC amplitudes were normalized to the first evoked cEPSC of each cell; error bars indicate standard error between cells. Inlays: Typical EPSC traces evoked by the specified stimulus. *Top: *Stimulation with 100 single pulses did not induce synaptic depression. The means of the first ten and last ten responses were not significantly different. *Middle: *Stimulation with 100 double pulses also failed to reduce cEPSC amplitudes. *Bottom: *Stimulation with 100 triple pulses induced an initial small potentiation followed by a weak decay of synaptic responses to about 93 ± 3 % of the initial amplitude. The means of the first ten and last ten averaged EPSCs were significantly different. B: Maximum cEPSC amplitudes in PnC giant neurons evoked by 100 bursts applied presynaptically at 1 Hz (n = 37) and 0.1 Hz (n = 10) to auditory fibers. Error bars indicate standard error between cells. All cEPSC amplitudes were normalized to the first evoked cEPSC of each cell. During 0.1 Hz stimulation, the cEPSC amplitudes declined to a plateau with about 64% of the initial amplitude. C: Amount of synaptic depression (in percent) induced by varying numbers of pulses within one burst applied to auditory fibers in rat brain slices at 1 Hz (*diamonds*) or 0.1 Hz (*triangle*). One and two pulse bursts did not induce synaptic depression while 100 triple pulses resulted in a small but significant decay. Four pulse bursts applied at 1 or 0.1 Hz induced synaptic depression of about 40% of the initial amplitude; stars indicate a significant decay.

As reported above, the application of paired pulses induced no synaptic depression but burst stimulation did. We thus examined in more detail which type of stimulus is required to induce synaptic depression. We truncated the bursts to only one, two or three pulses to see whether multiple high frequency stimuli are necessary to induce depression in the auditory pathway. Application of 100 single or double pulses with 4 ms ISI at 1 Hz did not yield any synaptic depression (Fig. 6A). There was no significant difference between the amplitudes of the first and the last ten averaged EPSCs (t(18) = 0.6, p = 0.27 for single pulses; t(20) = -0.9; p = 0.18 for double pulses, Fig. 6A). Stimulation with 100 triple pulses first led to a small potentiation, followed by a significant decay of response amplitudes to 93 ± 3 % (t(18) = 6.8; p < 0.0001, Fig. 6A). To summarize, synaptic depression strongly depended on repetitive stimulation by bursts; only bursts containing three or more pulses were able to induce synaptic depression.

### Evidence for a presynaptic mechanism

**Figure 7:**
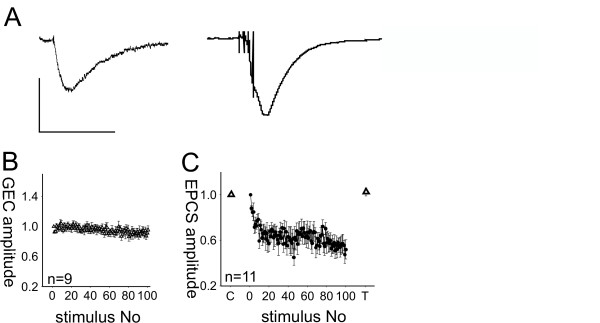
**Application of exogenous glutamate does not lead to receptor desensitization**. A: Currents in PnC giant neurons evoked by local uncaging of CNB-caged glutamate with a 10 μs UV-flash (*left*) and by presynaptic burst stimulation of auditory fibers in rat brain slices (*right*). Vertical scale bar corresponds to 300 pA, horizontal scalebar to 50 ms. B: Averaged amplitudes of glutamate-evoked currents (GECs, *white triangles*) evoked by photolysis of caged glutamate around the giant neuron soma under examination. 100 glutamate applications at 1 Hz produced no change in current amplitude; the means of the first ten and the last ten amplitudes (n = 9 PnC giant neurons) were not significantly different. Error bars indicate S.E.M. between cells. C: Averaged glutamate evoked currents (20 repetitions at 0.1 Hz per cell), before (control: C) and during synaptic depression (test: T). GECs were normalized to the average GEC of each cell under control conditions, since absolute amplitudes differed between cells. Synaptic depression was induced using a sequence of 100 bursts applied to auditory afferent fibers (*black circles*). cEPSC amplitudes were normalized to the first evoked cEPSC. Glutamate evoked currents did not change during synaptic depression (n = 11). Error bars indicate S.E.M.

The decrease in paired pulse ratio during depression and the need for repeated high frequency stimulation to produce depression both indicate that postsynaptic receptor desensitization may play a role in synaptic depression. If synaptic depression is indeed caused by receptor desensitization, longer intervals between bursts should decrease the amount of depression should be weaker with longer burst intervals; we therefore, we increased the interval between the bursts from one to ten seconds in another experiment. 100 bursts applied to auditory afferents at 0.1 Hz resulted in synaptic depression of the burst responses, reducing them to 64 ± 6 % (n = 8) with a course of depression similar to that induced by 1 Hz burst stimulation (Fig. 6B). There was no significant difference between the means of EPSCs 91–100 with stimulation at 1 Hz and 0.1 Hz (t(40) = 0.58, p = 0.56), which indicates that synaptic depression was independent from the burst intervals used. The degree of synaptic depression induced by different stimulus paradigms is summarized in figure 6C. Although the results again indicated that synaptic depression is the result of a presynaptic process, we wanted to look for more evidence that could exclude receptor desensitization as the mechanism underlying the depression. We combined presynaptic stimulation of auditory afferent fibers with the application of exogenous glutamate to PnC giant neurons in rat brain slices; the glutamate was delivered via focal glutamate uncaging, thus circumventing the presynaptic terminal. We adjusted the amount of glutamate uncaging in such a way that glutamate-evoked currents displayed time courses comparable to presynaptically evoked cEPSCs (Fig. 7A). The experiment revealed two main results; first, a sequence of 100 applications of exogenous glutamate at 1 Hz did not result in a decrease of glutamate-evoked current amplitude (-134.5 ± 42.1 pA for the first and -133.5 ± 43.8 pA for the 100^th ^glutamate-evoked current amplitude, t(8) = 1.24, p = 0.125, Fig. 7B). This indicates that repeated activation of glutamate receptors on PnC giant neurons at 1 Hz does not lead to receptor desensitization. Second, glutamate-evoked currents (GECs) in PnC giant neurons were measured before and immediately after depression was induced with a sequence of 100 burst stimuli applied to auditory afferent fibers. The amplitude and time course of the glutamate-evoked currents did not change during synaptic depression (-34.4 ± 9.8 pA before and -35.9.9 ± 9.8 pA after induction, t(10) = -1.4, p = 0.19, Fig. 7C), suggesting that no glutamate receptor desensitization occured. In summary, our data provided no evidence that postsynaptic glutamate receptor desensitization is the mechanism underlying synaptic depression in the PnC.

**Figure 8:**
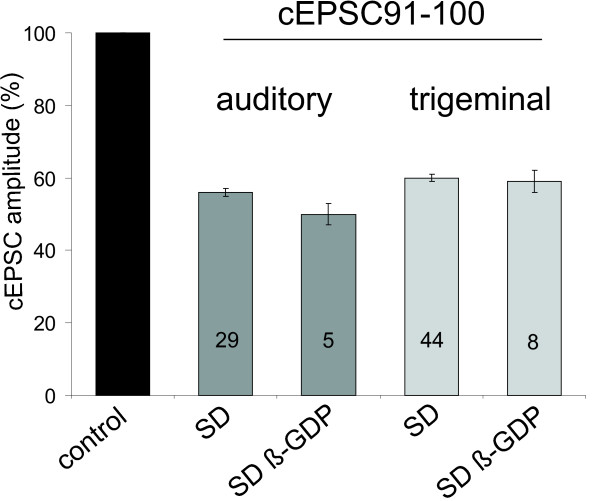
**Postsynaptic G-protein inactivation does not inhibit synaptic depression**. 100 bursts were applied to auditory or trigeminal afferent fibers. Compared to cEPSC1 (control), the burst sequences induced synaptic depression to about 60 % of initial amplitude (SD). This was also true for giant neurons that had been perfused with β-GDP using the patch pipette (SD β-GDP). cEPSC 91–100 were averaged for each cell, the number of cells is indicated for each group. Error bars indicate the standard error between cells.

Further evidence for a presynaptic depression mechanism was provided by experiments in which β-GDP (2 mM) was added to the patch pipette solution. β-GDP deactivates postsynaptic G-proteins upon diffusion into the recorded cell. The effect of β-GDP is especially interesting, since there is evidence for the involvement of metabotropic glutamate receptors (probably of group III) in the synaptic depression process [28]. The addition of β-GDP had no effect on synaptic depression in the auditory or trigeminal pathway; cEPSC amplitudes elicited by auditory or trigeminal afferents significantly decreased to 50 ± 3 % (auditory) and 58 ± 3 % (trigeminal) of the initial amplitude (general linear model, F(1,784) = 412, p < 0.0001 for auditory and F(1,791) = 439, p < 0.0001 for trigeminal cEPSCs, Fig. 8). The results show that no G-protein activation at postsynaptic sites is required for synaptic depression.

As a whole, our results provided an abundance of evidence that presynaptically-located synaptic depression in the PnC is the mechanism responsible for short-term habituation of the startle response.

## Discussion

In this study, auditory and trigeminal synapses in the PnC were stimulated presynaptically by applying short, high frequency bursts to afferent fibers of secondary neurons in the respective sensory pathway. We chose the burst stimulus paradigm as a model for the *in vivo *activity of auditory neurons during an acoustic startle experiment. Primary and secondary auditory neurons have been shown to fire at high frequency during sound presentation [29-31] and our burst duration resembles the duration of a startle stimulus. Giant neurons have a slow membrane time constant, enabling them to integrate repetitive auditory inputs as well as other sensory or modulatory input arriving at the neuron at different latencies [32]. Presynaptic bursts of action potentials produce compound synaptic currents whose amplitudes determine the probability that spiking will occur and influence the number of action potentials generated by PnC giant neurons [18]. Such compound excitatory synaptic currents (cEPSCs) are therefore a decisive factor for the elicitation and amplitude of a startle response.

In the first two experiments, we showed that in C57/Bl6 mice (and to some extent in rats) both the tactile and acoustic startle responses decreased when repetitive stimuli of these modalities were presented and similarly demonstrated that synaptic depression is induced when acoustic or trigeminal fibers synapsing on PnC giant neurons are repeatedly stimulated.

There was a slight difference in absolute acoustic startle amplitudes in one group of animals tested before tactile habituation (Fig. 4A, block 1–10) and in two other groups of animals tested either after tactile habituation or after exposure to the 95 dB background noise only (Fig. 4B, block 11–15). This difference was small (180 and 150 mV, respectively) and statistically insignificant. The 95 dB background noise used during tactile stimulation could account for the slightly lower acoustic startle amplitudes in Fig. 4B. However, after exposure to background noise, both groups of animals in Fig. 4B still showed acoustic startle habituation not statistically different from the habituation in Fig. 4A. The critical factor in this experiment is the lack of difference between the acoustic startle amplitudes of the two groups of mice in Fig. 4B (blocks 11–15, t-values < 1), even though only one group had received prior tactile stimulation.

Interestingly, the decline in startle response amplitudes of the C57BL/6 mice was generally greater when the tactile (behavioral) or trigeminal (electrophysiological) afferents were stimulated than when acoustic/auditory fibers were stimulated. This implies that short-term habituation to acoustic stimuli is generally weaker in these animals compared to habituation to tactile stimuli. Indeed, it has been reported that the amount of short-term habituation varies enormously between different stimulus modalities and that the degree of habituation is also dependent on the mouse strain [7]. However, we cannot completely exclude the possibility that habituation to the (perhaps fear and/or stress-inducing) background noise during tactile startle measurements summates with the tactile startle habituation, although the background noise was switched on 5 min. before measurements started and tactile habituation showed a perfect exponential decay. Moreover, background noise should actually lead to sensitization, a process that counteracts habituation [33]. Additionally, the behavioral results closely parallel the electrophysiological findings, adding to the growing body of evidence that PnC giant neurons are indeed a vital station in the startle pathway [13,16,18-20]. The results also strongly indicate that habituation takes place inside this pathway, namely in the form of synaptic depression at the synapse of the sensory inputs to the giant neurons [5,11,25-28].

### Localization of synaptic depression

The main goal of this study was to determine whether the cellular substrate for short-term habituation, which is presumably the result of synaptic depression in sensory synapses within the PnC, is located in the sensory part of the startle pathway (i.e. at the presynaptic terminals of sensory afferents), or if this is instead a postsynaptic feature. Given the association of synaptic depression with short-term habituation, a presynaptic mechanism would imply that short-term habituation occurs before signals from different pathways are integrated in the PnC. Habituation would therefore be specific for each stimulus modality and would not generalize between different modalities. We tested this in electrophysiological experiments with rats and mice and in behavioral experiments conducted with mice from the same strain (i.e. C57BL/6 mice, also commonly used to generate knock-out mice). This is vital, as large differences between different mouse strains have been found with respect to habituation [7,8].

Both approaches showed that both synaptic depression and short-term habituation are pathway specific. This is also consistent with behavioral studies of other species and mouse strains, which demonstrated that short-term habituation is modality specific [7,34]. The pathway specificity signals that a presynaptic mechanism is involved and further evidence is provided by our data showing that the paired pulse ratio changes during depression, since alterations in short-term plasticity are commonly thought to reflect presynaptic changes in the transmitter release machinery [34,35]. However, it should be noted that the paired pulse ratio declined during synaptic depression, which is the opposite of what would be expected if the decrease in synaptic efficacy is due to a reduced probability of transmitter release. This is normally accompanied by an increase in the paired pulse ratio, since the accumulated calcium in the presynaptic terminal meets a larger pool of releasable transmitter [35,36].

Different mechanisms could account for a reduced paired pulse ratio; either the desensitization of postsynaptic glutamate receptors or presynaptic mechanisms, such as the exhaustion of releasable transmitter in the presynaptic terminal.

### Mechanism of depression

Only repeated activation by short trains of at least four presynaptic action potentials produced an exponential decay of the cEPSC amplitudes in PnC neurons. Since trains of presynaptic action potentials lead to a strong and sustained release of the neurotransmitter glutamate into the synaptic cleft, desensitization of postsynaptic glutamate receptors could easily account for the resulting synaptic depression. However, several results rule out receptor desensitization as the underlying mechanism; first, the time course and the degree of depression were comparable when bursts were applied either every second or only every 10 seconds. In contrast, recovery from receptor desensitization should occur completely within 10 sec. Second, synaptic responses to single pulses as well as the responses to the first pulse within a burst were not (or not equally) affected by synaptic depression, but desensitized postsynaptic receptors should have had a similar effect on both response types. Moreover, repeated uncaging of exogenous glutamate at 1 Hz evoked responses with no sign of desensitization.

In summary, all of our results clearly contradict the hypothesis that postsynaptic receptor desensitization or changes in postsynaptic passive conductances can account for synaptic depression. Instead, our data provide ample behavioral and electrophysiological evidence that synaptic depression is a presynaptic phenomenon. Future studies will have to identify the molecular processes responsible for the alterations in synaptic transmission described here.

## Conclusion

We can conclude from our behavioral and electrophysiological data that synaptic depression and its behavioral correlate short-term habituation are features of the sensory branch of the startle pathway; specifically, they are a function of sensory synaptic terminals in the PnC. Synaptic depression/short-term habituation therefore occur prior to multimodal signal integration in the PnC, where signals from different pathways converge. Our data also further corroborate the association of short-term habituation and synaptic depression in the PnC.

## Methods

### Preparation of brain slices

Details of the preparation have been reported previously [28]. In brief, Sprague-Dawley rats or C57BL/6 mice (P10–14, Charles River, Sulzfeld, Germany) were deeply anesthetized with halothane, decapitated, and the brains were rapidly removed and transferred into ice-cold preparation solution containing (in mM): 210 sucrose, 26 NaHCO_3_, 1.3 MgSO_4_, 1.2 KH_2_PO_4_, 2 MgCl_2_, 2 KCl, 2 CaCl_2_, 10 glucose, 3 myoinositol, 2 sodium-pyruvate, 0.4 ascorbic acid, and equilibrated with 95% O_2_/5% CO_2_. Coronal slices (400 μm) were then cut with a vibratome (HM 650 V, Microm, Germany) in a submerged chamber filled with ice-cold preparation solution. Slices were transferred into a holding chamber filled with ACSF containing (in mM): 124 NaCl, 26 NaHCO_3_, 1.2 KH_2_PO_4_, 1.3 MgSO_4_, 2 KCl and 10 glucose. CaCl_2 _(2 mM) was added a few minutes after the slices had been transferred. The holding chamber was heated for 1 h to 34°, after which slices were kept at room temperature. All experiments were carried out in accordance with German and European animal protection laws.

### Electrophysiological recording

For recording, slices were transferred into a superfusion-recording chamber mounted on an upright, fixed stage microscope (Zeiss, Oberkochen, Germany) with infrared differential interference optics. Superfusion rate was 2–3 ml ACSF/min. Patch-clamp recordings were made at room temperature under visual guidance with an infrared-sensitive camera (Kappa, Germany). Patch electrodes were pulled from borosilicate capillaries (Science products, Hofheim, Germany) and filled with a solution containing (in mM): 130 potassium gluconate, 5 KCl, 0.6 EGTA, 10 HEPES, 2 MgCl_2_, pH 7.2 (KOH), 270–290 mOsm. Electrodes had a resistance of 2–5 MΩ. Giant neurons in the area of the PnC were identified by a soma diameter greater than 35 μm. All measurements were made in the voltage clamp configuration at -70 mV. Presynaptic stimuli were applied with bipolar concentric tungsten electrodes (SNEX, Science Products, Hofheim, Germany) connected to a stimulator (Isostim A320, wpi, Berlin, Germany). The stimulation electrodes were positioned in the area ventrolateral to the lateral superior olive (LSO) to stimulate auditory fibers arising from the cochlear nucleus and the cochlear root (see [28] for details and Fig. 2), and medial to the principal nucleus V (Pr5) in order to stimulate trigeminal fibers crossing from the Pr5 to the PnC (see [22,33] and Fig. 2). Recordings were made using an Axopatch 200 B amplifier and digitized with a Digidata 1320 (both Axon Instruments, Union City, USA). The data were filtered with a 10 kHz low-pass filter with a sampling rate of 20 kHz. pClamp 8.0.2 software (Axon Instruments, Union City, USA) was used for data acquisition and analysis. Stimulus intensities were kept low to avoid spiking of the neurons. Stimulus duration was 150 μs and synaptic depression was induced by a sequence of 100 burst stimuli at 1 Hz (unless otherwise noted). One burst consisted of four stimuli given within 12 ms (spacing 4 ms). Paired pulses were applied with a 50 ms interstimulus interval (ISI). Measurements were repeated ten times at 0.2 Hz for each cell before (control) and during synaptic depression induced by a sequence of 100 bursts. Ten traces were averaged for each condition and the paired pulse ratio was determined for each cell from the average trace. At least 5 min. recovery time was always allowed before applying a sequence of 100 bursts. The measurements under synaptic depression conditions followed immediately after the end of the 100 burst sequence. Maximum cEPSC amplitudes were measured (pA) and values were expressed as means ± SEM. Access resistance and seal quality was monitored at the beginning and several times during recordings to assure constant measurement conditions. Recordings were discarded when access resistance was larger than 30 MΩ or leak current more than -300 pA, or when one of these parameters changed by more than 15% during recording.

### Photolysis of caged glutamate

For the photolysis experiments, 5 mg of caged L-glutamic acid, (γ-CNB-caged L-glutamic acid, Molecular Probes, Leiden, the Netherlands) was dissolved in 10 ml oxygenated ACSF, corresponding to a concentration of 1.14 mM. The high concentration was chosen to avoid bleaching during 1 Hz photolysis. The solution was fed into a microcircuit perfusing the slice. The entire experimental setup was kept in a dark room illuminated only by flat screen computer monitors. Single UV flashes of ca. 10 μs duration and with 0–6 mJ energy were applied by a Flash Mic system (Rapp Opto Electronic, Hamburg, Germany), which was mounted on the microscope's epifluorescent optical pathway. The area of the slice exposed to the flash could be controlled by the aperture of the optical pathway; the soma of the recorded neuron was centered in the visual field and the aperture was set in such a way that the soma and the surrounding area of the PnC giant neuron under investigation were excited by photolyzed glutamate (size of illuminated spot: 150 μm diameter). Under the conditions described, CNB-caged glutamate had no effect on membrane potential, input resistance or spontaneous activity. CNB-caged glutamate containing ASCF could be used for two subsequent days without any sign of bleaching when stored in the dark overnight at 4°C.

### Behavioral experiments

Twelve naïve female C57BL/6 mice were obtained from Charles River, Sulzfeld. Mice were 7–8 weeks old at the beginning of the experiments. The mice were housed in groups of five in cages containing nesting material under a 12:12 hour light/dark schedule (lights on at 6 a.m.) and received food and tap water ad libidum. The cages were in an air-conditioned room (temperature: 24 ± 1°C, humidity: 60 ± 5%). The mice were adapted to the colony room for 14 days before testing began and all testing took place during the light period.

The apparatus for measurements of startle responses is described in detail elsewhere [7]. In short, the startle responses were measured inside a sound-attenuated chamber using a wire mesh test cage (5 × 9 × 5 cm) that was mounted on a movement-sensitive piezo accelerometer platform (Startle-Messsytem, University of Tübingen). Movement-induced voltage changes were digitized (Microstar, DAP1200e). Startle amplitudes were calculated as the difference between peak-to-peak voltage during a time window of 80 ms after stimulus onset and peak-to-peak voltage in the 80 ms time window before stimulus onset.

The tactile stimuli were air puffs of 100 Pascal (measured at the center of the cage; the air pressure before the air valve solenoid was 1.5 bar) delivered through a PVC tube centered on the side of the test cage; the distance to the center of the test cage was 8 cm. The air puff characteristics were measured using a 1-inch microphone (Bruel & Kjaer, model 4145). The air puffs had a duration of 30 ms, plus a rise time of 8 ms and a decay time of about 40 ms. Because the air puffs were given in the time window where startle was measured, they caused a "startle" artifact of 17 mV in the mean if measured with a weight simulating a mouse in the cage. In order to reduce noise generated by the air valve solenoid, the air passed through a "silencer" (see [7]). In order to mask the sound of the air puff itself, all testing of the tactile startle response was performed with background noise containing frequencies between 250 Hz and 20 kHz, with maximum intensity at 2 kHz. The noise was produced by a DSP-controlled system (Medav: Elf-Board with Siggen Software), amplified and emitted by a loudspeaker (Craaft HT 1640) inside the sound-absorbing chamber. The background noise level was 95 dB SPL RMS ("root mean square"), which completely masked the sound of the airpuff.

Acoustic stimuli were 14 kHz tones of 20 ms duration including 0.4 ms rise-/decay times. The SPL of these stimuli were 105 dB. The intertrial interval of acoustic and tactile stimuli was 15 sec.

The mice were adapted to the experimental environment inside the sound-attenuated chamber for 5 minutes on two days preceding testing. During this adaptation, background noise remained steady at 75 dB SPL RMS. On each test day, the mice were additionally allowed to adapt for 5 minutes before testing began; the following four test conditions were used: 1) "tactile→acoustic": 5 min adaptation (background noise 95 dB SPL) followed by 100 tactile stimuli (background noise 95 dB SPL) followed by 50 acoustic stimuli (background noise 50 dB SPL). 2) "acoustic only": no tactile stimuli (30 min background noise 95 dB SPL) followed by 50 acoustic stimuli (background noise 50 dB SPL). 3) "acoustic→tactile": 5 min adaptation (background noise 50 dB SPL) followed by 100 acoustic stimuli (background noise 50 dB SPL) followed by 50 tactile stimuli (background noise 95 dB SPL). 4) "tactile only": no acoustic stimuli (30 min background noise 50 dB SPL) followed by 50 tactile stimuli (background noise 95 dB SPL). Over four days, each mouse was tested once in each of the four conditions in a pseudorandom order.

For each block of 10 stimuli and each mouse, the mean of the ten startle responses was calculated; these means were used to calculate parametric statistics (grand mean and SEM). To test whether the startle response to the first 100 stimuli habituates, a general linear model was calculated using the block mean values (block as continuous factor, mouse as nominal factor of repeated measures). To test whether the startle response to the last 50 stimuli was dependent on the preceding treatment, t-tests were calculated.

## Authors' contributions

**Susanne Schmid: **Conception and Coordination of study. Acquisition of funding. Composition of the manuscript.

**Maruschka Weber, Nadine Simons, Claudia Plappert, Peter Pilz: **Acquisition of data, revision of manuscript
